# Targeting the Subventricular Zone to Promote Myelin Repair in the Aging Brain

**DOI:** 10.3390/cells11111809

**Published:** 2022-05-31

**Authors:** Arthur Morgan Butt, Andrea Dominico Rivera, Daniel Fulton, Kasum Azim

**Affiliations:** 1 Institute of Biomedical and Biomolecular Sciences, School of Pharmacy and Biomedical Science, University of Portsmouth, St. Michael’s Building, White Swan Road, Portsmouth PO1 2DT, UK; andrea.rivera@unipd.it; 2 Section of Human Anatomy, Department of Neuroscience, University of Padua, 35122 Padua, Italy; 3 Neuroscience and Ophthalmology Research Group, Institute of Inflammation and Ageing, College of Medical and Dental Sciences, University of Birmingham, Birmingham B15 2TT, UK; d.fulton@bham.ac.uk; 4 Department of Neurology, Medical Faculty, Heinrich-Heine-University, 40225 Düsseldorf, Germany

**Keywords:** oligodendrogenesis, subventricular zone, aging, multiple sclerosis, remyelination

## Abstract

The subventricular zone (SVZ) is the largest and most active germinal zone in the adult forebrain. Neural stem cells (NSCs) of the SVZ generate olfactory interneurons throughout life and retain the intrinsic ability to generate oligodendrocytes (OLs), the myelinating cells of the central nervous system. OLs and myelin are targets in demyelinating diseases such as multiple sclerosis (MS). Remyelination is dependent on the ability of oligodendrocyte progenitor cells (OPCs) to proliferate, migrate, and terminally differentiate into myelinating OLs. During aging, there is a gradual decrease in the regenerative capacity of OPCs, and the consequent loss of OLs and myelin is a contributing factor in cognitive decline and the failure of remyelination in MS and other pathologies with aging contexts, including Alzheimer’s disease (AD) and stroke. The age-related decrease in oligodendrogenesis has not been fully characterised but is known to reflect changes in intrinsic and environmental factors affecting the ability of OPCs to respond to pro-differentiation stimuli. Notably, SVZ-derived OPCs are an important source of remyelinating OLs in addition to parenchymal OPCs. In this mini-review, we briefly discuss differences between SVZ-derived and parenchymal OPCs in their responses to demyelination and highlight challenges associated with their study in vivo and how they can be targeted for regenerative therapies in the aged brain.

## 1. CNS (re)Myelination Efficiency Declines Significantly with Age

In the central nervous system (CNS), oligodendrocytes (OLs) enable rapid axonal conduction of electrical impulses by producing myelin, a lipid-rich membrane that acts as an axonal insulator and contributes to their metabolic support [1]. In both mice and humans, the bulk of oligodendrogenesis and thus myelin deposition takes place by oligodendrocyte progenitor cells (OPCs) during adolescence and young adulthood, but the addition of newly generated OLs and myelin replenishment are life-long processes [2]. Importantly, OPCs persist throughout the parenchyma of the adult brain and spinal cord and are committed to the life-long generation of OLs, which is a dynamic process that can be modulated to meet local requirements, such as myelin remodelling in response to changes in neuronal activity or myelin loss due to pathology (reviewed extensively elsewhere, for example [3]). By a process of self-replication, OPCs generate OLs by differentiation along the lineage whilst maintaining a relatively stable population of slowly proliferating parenchymal OPCs [4,5,6]. Compensatory OPC proliferation and differentiation are rapidly induced in response to loss of oligodendroglial lineage cells, ensuring both OPC population homeostasis and myelin repair. However, despite relatively stable densities of parenchymal OPCs, oligodendrogenesis declines with age, at least in part due to decreasing responsiveness to pro-differentiation signals [3,7,8]. Other physiological functions of OPCs also undergo age-related deterioration, such as the regulation of neurotransmission [9,10] and potentially the maintenance of homeostatic microglial phenotypes [11]. The reduced ability of aged OPCs to respond to and compensate for myelin loss results in the ultimate failure of effective remyelination, as observed in relapse-remitting multiple sclerosis (MS), which is characterised by efficient remyelination (remittance) in younger individuals, and very often secondary progressive MS at later stages of the disease. Notably, there is accumulating evidence that OPCs derived from neural stem cells (NSCs) of the subventricular zone (SVZ) play a major role in replenishing parenchymal OPCs and supporting myelin repair in the forebrain. In this mini-review, we highlight how the SVZ can be targeted therapeutically to stimulate OPC recruitment and promote remyelination in the aging brain.

## 2. Oligodendrogenesis in the SVZ Is Spatially and Temporally Conserved across Species

**Figure 1:**
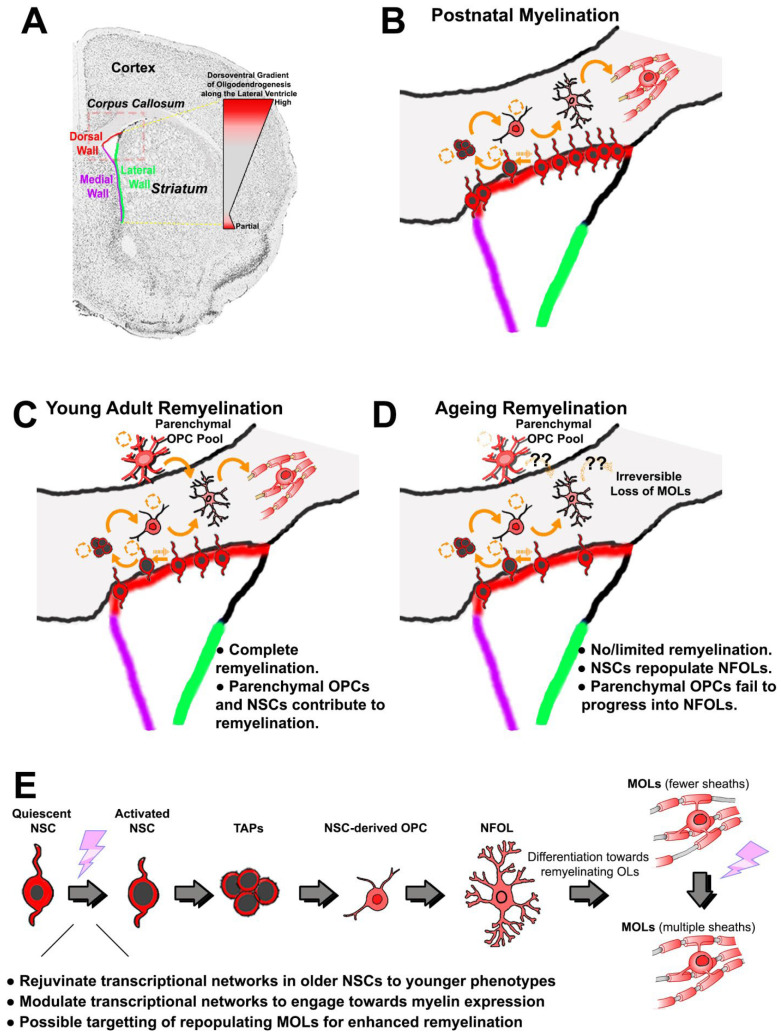
Forebrain oligodendrogenesis and remyelination efficiencies in young versus aged adults. (**A**) Coronal brain section counterstained for nuclei; the corpus callosum is evident as light grey, and the SVZ zones and other regions of interest are indicated. The dorsoventral gradient of oligodendrogenesis in the SVZ is illustrated; OPCs are generated primarily from NSCs in the dorsal microdomain, and at lower rates in the most ventral regions of the SVZ. This preferential generation of OLs from the dorsal SVZ persists in adulthood and is increased following demyelination. (**B**) During postnatal development, the majority of OLs in the dorsal forebrain are derived from NSC located in the dorsal SVZ that progress through a number of distinct differentiation stages in response to intrinsic and extrinsic cues (see (E) for explanations of pictograms of the differentiation stages): quiescent NSCs have small nuclei and in response to appropriate stimuli can transform into activated NSCs that have larger nuclei; activated NSCs generate transiently amplifying progenitors (TAPs), which is a pre-OPC stage that gives rise to migratory and proliferative OPCs with a simple processing-bearing morphology; OPCs migrate to their final sites, where they undergo self-replication and generate newly formed (NF)OLs, which have a complex process-bearing morphology and are non-proliferative; NFOLs differentiate into mature myelinating (M)OLs; slowly proliferating parenchymal OPCs with a highly complex ramified morphology persist after the main developmental period of myelination. (**C**) In young adults, demyelinating insults trigger efficient remyelination by parenchymal OPCs that are located at or near to the lesion site. Additionally, morphologically simpler and highly migratory OPCs are recruited from dorsal NSCs of the SVZ to replenish parenchymal OPCs and contribute to remyelination. (**D**) The aged brain is characterised by inefficient regeneration of MOLs both from parenchymal and SVZ-derived OPCs, resulting in impaired remyelination; in the aged SVZ, dorsal NSCs are able to regenerate NFOLs, but these fail to progress into remyelinating MOLs, suggesting a deficiency of appropriate extrinsic stimuli (indicated by ‘?’). (**E**) Identifying the transcriptional networks that regulate each stage of oligodendrogenesis from dorsal NSCs will enable the development of targeted therapies that rejuvenate aged NSCs and stimulate replenishment of OPCs to promote remyelination and repair in the aged brain. Abbreviations: NSC = neural stem cell; TAPs = transiently amplifying progenitors (pre-OPC stage); OPC = oligodendrocyte precursor cell; NFOL = non-myelin forming oligodendrocyte; MOL = mature oligodendrocyte.

The majority of OLs found in the adult mouse forebrain originate postnatally from neural stem cells (NSCs) that reside in the subventricular zone (SVZ), via a defined series of differentiation steps (see reviews [12,13] (Figure 1A,B)), and it is established that the early postnatal period is critical for OL specification and myelination [14,15,16]. Notably, the murine SVZ is spatially heterogeneous and contains specific microdomains of NSCs that are biased to give rise to defined lineages, depending on intrinsic and extrinsic factors, with OPCs being derived primarily from NSCs located in the dorsal SVZ [17,18]. Importantly, studies performed by Zecevic and co-workers have demonstrated an equivalent spatial organisation of human SVZ-NSCs during early development, where oligodendrogenesis emerges largely from more dorsal NSC subpopulations [19,20]. More recent studies have confirmed these findings in primates, guided by the expression of pallial transcriptional cues in NSCs such as *Hopx* [21], and humans, where radial glia switch developmentally from neurogenesis to oligodendrogenesis [22]. Thus, it is apparent that SVZ microdomains observed in the mouse are representative of species with more complex brain architectures, including humans [19,20,23]. This organisation persists after postnatal development and is important for adult OL regeneration [24,25,26,27,28]. Single-cell RNA sequencing (ScRNA-seq), coupled with long-term genetic fate mapping approaches, have characterised NSCs residing within the most lateral and ventral aspects of the rodent SVZ and show that they become increasingly quiescent during aging [29,30,31,32]. Analyses of aged dorsal NSCs at the single-cell RNA level are still lacking, but meta-analyses of published datasets support their life-long persistence and indicate they are largely quiescent under physiological conditions but can be stimulated in pathological contexts [32,33]. In humans, NSCs identified by their expression of β4 tubulin persist after postnatal development in all domains of the SVZ and, although they do not express most markers that are used to identify rodent NSCs, human NSCs do however appear to express GFAP-delta and cell surface receptors responsive to FGF2 and EGF, as in rodent NSCs [34,35,36]. Moreover, a recent elegant ScRNA-seq study using the broad progenitor marker CD271 demonstrated NSCs persist in the human dorsal SVZ of healthy individuals aged between 72 and 96 years [37]. Meta-analysis and integration with single cell datasets of human oligodendroglia [38], revealed that these dorsal SVZ-derived NSCs have a pro-oligodendroglial phenotype.

The causes of age-related quiescence of SVZ-NSC and their progeny are unresolved, but there is evidence of an important role for canonical Wnt signalling, which maintains the dorsal identity of the SVZ throughout life in the mammalian brain (reviewed in [12]). The aged SVZ expresses the inhibitory ligands secreted frizzled-related proteins (SFRPs), which are potent inhibitors of the canonical Wnt pathway and limit both neurogenesis and oligodendrogenesis [32,38]. Attenuation of SFRPs in human IPSCs lines [38] and mouse models of demyelination [39] corroborate that repression of canonical Wnt signalling is an important factor in driving SVZ-NSCs and their progeny towards oligodendrogenesis. These studies amongst others confirm that life-long oligodendrogenesis occurs in the human SVZ, but progress in our understanding of these processes has been limited by several challenges, not least the difficulty of systematic sampling of human tissue due to the large area to cover and the challenge of assessing current gold-standard markers with high confidence on preserved human tissue. Furthermore, as noted above, the antigenicity of human and rodent SVZ-derived NSC differs considerably, and defined markers that distinguish human NSC from astrocytic lineages are required to enable accurate studies of human populations in the context of brain aging, disease, and trauma.

## 3. Recruitment of SVZ NSCs for Oligodendroglial Replacement

Following myelin loss in the rodent CNS, it has been demonstrated that OPCs located within and adjacent to demyelinated lesions proliferate, migrate, and differentiate into remyelinating OLs [13,40]. This process is pronounced and efficient in early disease stages and in young adults (Figure 1C). OLs that persist within demyelinated lesions are also capable of remyelination [41,42,43,44] (reviewed in detail elsewhere, see, for example, [40]). Studies using ^14^C levels in humans concluded that surviving OLs rather than OPCs play the major role in remyelination in MS [41,42], except in very aggressive forms of the disease where OPCs are more important [45]. Our understanding of these processes comes from studies in rodent models, and fate-mapping studies demonstrate unequivocally that remyelination in the adult brain is from both parenchymal and SVZ-derived OPCs. However, in older rodents, recruitment of OPCs and their differentiation into MOLs is incomplete, involving multiple processes [7,9,46,47]. The ultimate failure of remyelination coupled to continuous loss of myelin with time is the basis of progressive disability in MS patients [41,42,43]. Consistent with this, studies of human post-mortem tissue have shown that OPCs are present in active and remyelinating lesions, and that they are stunted in chronic lesions. Thus, there is a clear and unmet need to develop new therapies to rejuvenate OPCs and stimulate remyelination in the aged brain at later stages of MS.

## 4. Utilising Mouse Modes of Demyelination with Greater Relevancy to Human MS

Our own recent observations, together with key studies by others, indicate that demyelination in mice aged from around 6 months of age provides a reasonable model of human aging, since the pace and extent of remyelination via SVZ-NSCs or parenchymal OPCs is already significantly reduced at this age compared to younger mice (see references [46,48] and thoroughly reviewed in [40]). Interestingly, this decline parallels a similar decrease in olfactory bulb neurogenesis, suggesting a progressive depletion of NSC or their entrance into a “deep-state” quiescence, whereby they are no longer committed to active or proliferative states [29,32]. How the decline in neurogenesis of the aging SVZ applies to the oligodendrogenic dorsal domains of the SVZ remains unclear. Interestingly, the transcriptomes of quiescent NSCs of 2-month-old versus 22-month-old mice are remarkably similar for at least lateral wall NSCs, with most transcriptional changes being observed in subpopulations of active NSCs [31,32]. We know from our own studies that the dorsal wall enters quiescence relatively early in adulthood at around 4 months of age, unlike the lateral wall, which retains a basal level of NSC activation long into aging [24,30]. Nevertheless, at least in the healthy brain, and as discussed below, quiescent dorsal NSCs can be reactivated by stimulating appropriate signalling pathways, which are abundant during postnatal development, resulting in dramatic oligodendrogenesis [24], suggestive of context-dependent plasticity.

Interestingly, distinct adult SVZ domains can be distinguished based on their age-associated response to demyelinating lesions [48,49,50]. Whilst oligodendrogenesis and remyelination lead to nearly complete myelin restoration in younger mice, from 6 months of age, there appears to be an age-related impairment in the generation of remyelinating OLs, despite large increases in all stages of the OL lineage up to the non-myelin-forming OLs (NFOL) stage [48,49,50,51,52]. In addition, NFOL differentiation from parenchymal OPCs during remyelination is decreased in mice beyond 6 month compared to NFOLs that were derived from dorsal NSCs [48] (see also Figure 1D). These findings underline that whilst NFOLs can be formed (Figure 1D), their differentiation into MOLs is impaired, which may reflect age-associated environmental conditions that are unfavourable to terminal differentiation and/or myelination. In contrast to neurogenesis (see for example, [31,32]), the nature of the age-related heterogeneity in the environmental factors and intracellular programs for OL lineage cells are currently poorly defined. It is anticipated that new studies in the field will shed light on the intrinsic temporal differences in oligodendrogenesis.

In the EAE (experimental autoimmune encephalomyelitis) mouse preclinical model of chronic demyelination provoked by inflammation, studies focusing on NSCs have been reliant on histological readouts rather than genetic fate-mapping. By virtue of reaching the chronic phase of disease (achieved after 2 months of EAE treatment beginning in the second or third month of life), treated mice are already at the onset of aging. In the few studies examining the SVZ in chronic EAE conditions, inflammation (namely, inhibitory cytokines and chemokines) drastically restricts the oligodendrogenic capacity of NSCs, although this is apparently readily reversed by anti-inflammatory compounds, facilitating regeneration [53,54,55]. The impact of chronic EAE on the SVZ has been examined on lateral and ventral SVZ NSCs, but it remains to be determined if, and to what degree, the reported findings apply to the dorsal SVZ. As described above, a recent finding from toxin models of demyelination in both younger and older mice reveal a greater efficiency for terminal OL differentiation among dorsal [48] versus parenchymal OPCs [48,49,51] (Figure 1E). Our own recent investigations into the age-related dysfunction of OPCs revealed transcriptional correlates of age-related changes in remyelinating potential [46]. Importantly, pharmacogenomic approaches can guide the therapeutic amelioration of this defect, inducing developmental transcriptional states associated with improved progression to remyelinating OLs [46,56]. Our findings strongly support the ability of sequential strategies, aimed first at “rejuvenating” aged OPCs, followed by additional stimuli for targeting downstream networks in terminally differentiated OLs to promote a robust remyelination response in the aged brain. These findings imply that the remyelination capacity of activated NSCs could be improved with a single exogenous stimulus (Figure 1E), while parenchymal OPCs may require more complex multi-therapeutic strategies, which could be more costly to develop and challenging to apply clinically. It is clear from the above studies that analysis of demyelination beyond younger adult ages (2–3 months) is required to provide important insights into later stages of MS in humans.

## 5. Intrinsic Differences in Remyelinating Pools

While environmental factors are likely to differ dramatically between younger and older mice, an additional issue of interest is that of potential intrinsic differences between resident OPCs and those generated from SVZ NSCs [48,49]. A key earlier study described how OPCs generated from NSCs migrate at a faster rate than parenchymal OPCs [57]. In addition, in vitro observations from 8-month-old mice brains show that OPCs derived from the dorsal SVZ exhibit enhanced migration compared to their ventral counterparts [48]. Another intriguing observation from fate-mapping studies in young adult mice demonstrated that following demyelination the g-ratios (indicative of myelin quantity per OL-axon unit) are better restored from OLs that were derived from NSCs (Nestin-CreERT2 fate mapping) compared to those generated via OPCs (Pdgfra-CreERT2 fate mapping) [49]. Thus, parenchymal OPCs and NSC-derived OPCs may have intrinsic differences in their differentiation potentials and responses to environmental factors. Further studies are required using SVZ-microdomain-specific Cre drivers to determine whether this holds true in aging mice. A recent transcriptomic analysis has characterised the changes between SVZ-derived Ascl+ pre-OPCs (also known as transiently amplifying progenitors (TAPs)) through to OPC stages in young adult mice [51], but the molecular basis of differences between SVZ-derived OPCs and parenchymal OPCs remains unclear. Specific lineage sampling approaches and ScRNA-seq profiling will be necessary to deepen our understanding of heterogeneity within the oligodendroglial lineage.

## 6. Summary and Outlook

In summary, OL differentiation via parenchymal OPCs is limited in the aged brain, and insights gained from comparative molecular profiling of OL lineage cells across ages indicate that these limitations can be circumvented by age- and stage-specific strategies to rejuvenate oligodendrogenesis. Furthermore, the possibility of greater oligodendrogenic potential of NSC-derived OPCs compared to parenchymal OPCs, in both aged and demyelinated settings, may be crucial to identify pharmacological strategies to either specifically boost oligodendrogenesis from the SVZ, or to endow parenchymal OPCs with attributes typical of their SVZ-derived counterparts (Figure 1E). In this respect, pharmacogenomic screening procedures are a powerful tool for identifying the most potent therapeutic agents and establishing optimal in vivo dosages [24,46,56,58]. As a next step to design more effective therapeutic strategies, high-throughput “omics” experiments and 3-dimensional ex vivo human SVZ models are needed. The presence of early glial progenitors in the aged human SVZ has been demonstrated in the healthy brain [38] and in a number of disease conditions, including MS [34,55,59], AD [60], and Parkinson’s disease (PD) [37]. Earlier studies using a limited set of SVZ-NSC markers in post-mortem tissue from human MS patients [34,55,59] clearly underline the complexities associated with their detection. A recent study by Donega and co-workers used the surface marker CD271 for isolating several thousand SVZ progenitors from control and PD patients [37], and a similar strategy could be used to isolate and sequence microdomain specific cells from the human SVZ from control and MS patients, perhaps by combining a surface marker such as CD271 with other markers to enrich specifically for NSCs, whilst also capturing additional cell types present in the niche. These experiments, combined with the results of recent landmark studies [41,42], can help unravel how individual stages of the OL lineage may be affected by age, depending on their origins (NSCs vs. parenchymal OPCs). Future studies to test rejuvenating therapies in human SVZ will require the development of suitable complex three-dimensional ex vivo models, for example, using hiPSC-derived organoids capable of replicating the structure and function of the adult human SVZ and adjacent white matter. In this regard, the advent of “myelinoids”, which to date best resemble the 3D topographical features of human white matter [61], and addition of an adjoining “germinal niche” would be of considerable benefit for devising NSC-directed therapies. In conclusion, our studies demonstrate that it is possible to control the fate of SVZ-derived NSCs in the mouse and identify novel therapeutics that promote remyelination in aging contexts, which is highly relevant to MS and other diseases.

## Data Availability

Not applicable.

## References

[1] Stadelmann C., Timmler S., Barrantes-Freer A., Simons M. (2019). Myelin in the Central Nervous System: Structure, Function, and Pathology. Physiol. Rev..

[2] Hill R.A., Li A.M., Grutzendler J. (2018). Lifelong cortical myelin plasticity and age-related degeneration in the live mammalian brain. Nat. Neurosci..

[3] Rivera A.D., Azim K., Macchi V., Porzionato A., Butt A.M., De Caro R. (2022). Epidermal Growth Factor Pathway in the Age-Related Decline of Oligodendrocyte Regeneration. Front. Cell. Neurosci..

[4] Butt A.M., Papanikolaou M., Rivera A. (2019). Physiology of Oligodendroglia. Adv. Exp. Med. Biol..

[5] Akay L.A., Effenberger A.H., Tsai L.H. (2021). Cell of all trades: Oligodendrocyte precursor cells in synaptic, vascular, and immune function. Genes Dev..

[6] Kula B., Chen T.J., Kukley M. (2019). Glutamatergic signaling between neurons and oligodendrocyte lineage cells: Is it synaptic or non-synaptic?. GLIA.

[7] Neumann B., Baror R., Zhao C., Segel M., Dietmann S., Rawji K.S., Foerster S., McClain C.R., Chalut K., van Wijngaarden P. (2019). Metformin Restores CNS Remyelination Capacity by Rejuvenating Aged Stem Cells. Cell Stem. Cell.

[8] Rivera A.D., Chacon-De-La-Rocha I., Pieropan F., Papanikolau M., Azim K., Butt A.M. (2021). Keeping the ageing brain wired: A role for purine signalling in regulating cellular metabolism in oligodendrocyte progenitors. Pflug. Arch. Eur. J. Physiol..

[9] Chacon-De-La-Rocha I., Fryatt G., Rivera A.D., Verkhratsky A., Raineteau O., Gomez-Nicola D., Butt A.M. (2020). Accelerated Dystrophy and Decay of Oligodendrocyte Precursor Cells in the APP/PS1 Model of Alzheimer’s-Like Pathology. Front. Cell. Neurosci..

[10] Vanzulli I., Papanikolaou M., De-La-Rocha I.C., Pieropan F., Rivera A.D., Gomez-Nicola D., Verkhratsky A., Rodriguez J.J., Butt A.M. (2020). Disruption of oligodendrocyte progenitor cells is an early sign of pathology in the triple transgenic mouse model of Alzheimer’s disease. Neurobiol. Aging.

[11] Liu Y., Aguzzi A. (2020). NG2 glia are required for maintaining microglia homeostatic state. GLIA.

[12] Azim K., Berninger B., Raineteau O. (2016). Mosaic Subventricular Origins of Forebrain Oligodendrogenesis. Front. Neurosci..

[13] El Waly B., Macchi M., Cayre M., Durbec P. (2014). Oligodendrogenesis in the normal and pathological central nervous system. Front. Neurosci..

[14] Liu R., Jia Y., Guo P., Jiang W., Bai R., Liu C. (2021). In Vivo Clonal Analysis Reveals Development Heterogeneity of Oligodendrocyte Precursor Cells Derived from Distinct Germinal Zones. Adv. Sci..

[15] Tong C.K., Fuentealba L.C., Shah J.K., Lindquist R.A., Ihrie R.A., Guinto C.D., Rodas-Rodriguez J.L., Alvarez-Buylla A. (2015). A Dorsal SHH-Dependent Domain in the V-SVZ Produces Large Numbers of Oligodendroglial Lineage Cells in the Postnatal Brain. Stem. Cell Rep..

[16] Kessaris N., Fogarty M., Iannarelli P., Grist M., Wegner M., Richardson W.D. (2006). Competing waves of oligodendrocytes in the forebrain and postnatal elimination of an embryonic lineage. Nat. Neurosci..

[17] Azim K., Hurtado-Chong A., Fischer B., Kumar N., Zweifel S., Taylor V., Raineteau O. (2015). Transcriptional Hallmarks of Heterogeneous Neural Stem Cell Niches of the Subventricular Zone. Stem. Cells.

[18] Azim K., Rivera A., Raineteau O., Butt A.M. (2014). GSK3beta regulates oligodendrogenesis in the dorsal microdomain of the subventricular zone via Wnt-beta-catenin signaling. GLIA.

[19] Rakic S., Zecevic N. (2003). Early oligodendrocyte progenitor cells in the human fetal telencephalon. GLIA.

[20] Jakovcevski I., Filipovic R., Mo Z., Rakic S., Zecevic N. (2009). Oligodendrocyte development and the onset of myelination in the human fetal brain. Front. Neuroanat..

[21] Rash B.G., Duque A., Morozov Y.M., Arellano J.I., Micali N., Rakic P. (2019). Gliogenesis in the outer subventricular zone promotes enlargement and gyrification of the primate cerebrum. Proc. Natl. Acad. Sci. USA.

[22] Fu Y., Yang M., Yu H., Wang Y., Wu X., Yong J., Mao Y., Cui Y., Fan X., Wen L. (2021). Heterogeneity of glial progenitor cells during the neurogenesis-to-gliogenesis switch in the developing human cerebral cortex. Cell Rep..

[23] Azim K., Zweifel S., Klaus F., Yoshikawa K., Amrein I., Raineteau O. (2013). Early decline in progenitor diversity in the marmoset lateral ventricle. Cereb Cortex.

[24] Azim K., Angonin D., Marcy G., Pieropan F., Rivera A., Donega V., Cantu C., Williams G., Berninger B., Butt A.M. (2017). Pharmacogenomic identification of small molecules for lineage specific manipulation of subventricular zone germinal activity. PLoS Biol..

[25] Azim K., Raineteau O., Butt A.M. (2012). Intraventricular injection of FGF-2 promotes generation of oligodendrocyte-lineage cells in the postnatal and adult forebrain. GLIA.

[26] Kang W., Nguyen K.C.Q., Hebert J.M. (2019). Transient Redirection of SVZ Stem Cells to Oligodendrogenesis by FGFR3 Activation Promotes Remyelination. Stem. Cell Rep..

[27] Embalabala R.J., Brockman A.A., Jurewicz A.R., Kong J.A., Ryan K., Guinto C.D., Alvarez-Buylla A., Chiang C., Ihrie R.A. (2022). GLI3 is Required for OLIG2+ Progeny Production in Adult Dorsal Neural Stem Cells. Cells.

[28] Vancamp P., Gothie J.D., Luongo C., Sebillot A., Le Blay K., Butruille L., Pagnin M., Richardson S.J., Demeneix B.A., Remaud S. (2019). Gender-specific effects of transthyretin on neural stem cell fate in the subventricular zone of the adult mouse. Sci. Rep..

[29] Bast L., Calzolari F., Strasser M.K., Hasenauer J., Theis F.J., Ninkovic J., Marr C. (2018). Increasing Neural Stem Cell Division Asymmetry and Quiescence Are Predicted to Contribute to the Age-Related Decline in Neurogenesis. Cell Rep..

[30] Calzolari F., Michel J., Baumgart E.V., Theis F., Gotz M., Ninkovic J. (2015). Fast clonal expansion and limited neural stem cell self-renewal in the adult subependymal zone. Nat. Neurosci..

[31] Dulken B.W., Buckley M.T., Negredo P.N., Saligrama N., Cayrol R., Leeman D.S., George B.M., Boutet S.C., Hebestreit K., Pluvinage J.V. (2019). Single-cell analysis reveals T cell infiltration in old neurogenic niches. Nature.

[32] Kalamakis G., Brune D., Ravichandran S., Bolz J., Fan W., Ziebell F., Stiehl T., Catala-Martinez F., Kupke J., Zhao S. (2019). Quiescence Modulates Stem Cell Maintenance and Regenerative Capacity in the Aging Brain. Cell.

[33] Borrett M.J., Innes B.T., Jeong D., Tahmasian N., Storer M.A., Bader G.D., Kaplan D.R., Miller F.D. (2020). Single-Cell Profiling Shows Murine Forebrain Neural Stem Cells Reacquire a Developmental State when Activated for Adult Neurogenesis. Cell Rep..

[34] Wu C., Chang A., Smith M.C., Won R., Yin X., Staugaitis S.M., Agamanolis D., Kidd G.J., Miller R.H., Trapp B.D. (2009). Beta4 tubulin identifies a primitive cell source for oligodendrocytes in the mammalian brain. J. Neurosci..

[35] Roelofs R.F., Fischer D.F., Houtman S.H., Sluijs J.A., Van Haren W., Van Leeuwen F.W., Hol E.M. (2005). Adult human subventricular, subgranular, and subpial zones contain astrocytes with a specialized intermediate filament cytoskeleton. GLIA.

[36] Van den Berge S.A., Middeldorp J., Zhang C.E., Curtis M.A., Leonard B.W., Mastroeni D., Voorn P., van de Berg W.D., Huitinga I., Hol E.M. (2010). Longterm quiescent cells in the aged human subventricular neurogenic system specifically express GFAP-delta. Aging Cell.

[37] Donega V., Burm S.M., van Strien M.E., van Bodegraven E.J., Paliukhovich I., Geut H., van de Berg W.D.J., Li K.W., Smit A.B., Basak O. (2019). Transcriptome and proteome profiling of neural stem cells from the human subventricular zone in Parkinson’s disease. Acta Neuropathol. Commun..

[38] Donega V., van der Geest A.T., Sluijs J.A., van Dijk R.E., Wang C.C., Basak O., Pasterkamp R.J., Hol E.M. (2022). Single-cell profiling of human subventricular zone progenitors identifies SFRP1 as a target to re-activate progenitors. Nat. Commun..

[39] Huang S., Choi M.H., Huang H., Wang X., Chang Y.C., Kim J.Y. (2020). Demyelination Regulates the Circadian Transcription Factor BMAL1 to Signal Adult Neural Stem Cells to Initiate Oligodendrogenesis. Cell Rep..

[40] Cayre M., Falque M., Mercier O., Magalon K., Durbec P. (2021). Myelin Repair: From Animal Models to Humans. Front. Cell. Neurosci..

[41] Jäkel S., Agirre E., Falcao A.M., van Bruggen D., Lee K.W., Knuesel I., Malhotra D., Ffrench-Constant C., Williams A., Castelo-Branco G. (2019). Altered human oligodendrocyte heterogeneity in multiple sclerosis. Nature.

[42] Falcao A.M., van Bruggen D., Marques S., Meijer M., Jäkel S., Agirre E., Samudyata, Floriddia E.M., Vanichkina D.P., Ffrench-Constant C. (2018). Disease-specific oligodendrocyte lineage cells arise in multiple sclerosis. Nat. Med..

[43] Patrikios P., Stadelmann C., Kutzelnigg A., Rauschka H., Schmidbauer M., Laursen H., Sorensen P.S., Bruck W., Lucchinetti C., Lassmann H. (2006). Remyelination is extensive in a subset of multiple sclerosis patients. Brain.

[44] Patani R., Balaratnam M., Vora A., Reynolds R. (2007). Remyelination can be extensive in multiple sclerosis despite a long disease course. Neuropathol. Appl. Neurobiol..

[45] Yeung M.S.Y., Djelloul M., Steiner E., Bernard S., Salehpour M., Possnert G., Brundin L., Frisen J. (2019). Dynamics of oligodendrocyte generation in multiple sclerosis. Nature.

[46] Rivera A.D., Pieropan F., Chacon-De-La-Rocha I., Lecca D., Abbracchio M.P., Azim K., Butt A.M. (2021). Functional genomic analyses highlight a shift in Gpr17-regulated cellular processes in oligodendrocyte progenitor cells and underlying myelin dysregulation in the aged mouse cerebrum. Aging Cell.

[47] Segel M., Neumann B., Hill M.F.E., Weber I.P., Viscomi C., Zhao C., Young A., Agley C.C., Thompson A.J., Gonzalez G.A. (2019). Author Correction: Niche stiffness underlies the ageing of central nervous system progenitor cells. Nature.

[48] Crawford A.H., Tripathi R.B., Richardson W.D., Franklin R.J. (2016). Developmental Origin of Oligodendrocyte Lineage Cells Determines Response to Demyelination and Susceptibility to Age-Associated Functional Decline. Cell Rep..

[49] Xing Y.L., Roth P.T., Stratton J.A., Chuang B.H., Danne J., Ellis S.L., Ng S.W., Kilpatrick T.J., Merson T.D. (2014). Adult neural precursor cells from the subventricular zone contribute significantly to oligodendrocyte regeneration and remyelination. J. Neurosci..

[50] Brousse B., Magalon K., Durbec P., Cayre M. (2015). Region and dynamic specificities of adult neural stem cells and oligodendrocyte precursors in myelin regeneration in the mouse brain. Biol. Open.

[51] Brousse B., Mercier O., Magalon K., Daian F., Durbec P., Cayre M. (2021). Endogenous neural stem cells modulate microglia and protect against demyelination. Stem. Cell Rep..

[52] Kazanis I., Evans K.A., Andreopoulou E., Dimitriou C., Koutsakis C., Karadottir R.T., Franklin R.J.M. (2017). Subependymal Zone-Derived Oligodendroblasts Respond to Focal Demyelination but Fail to Generate Myelin in Young and Aged Mice. Stem. Cell Rep..

[53] Pluchino S., Muzio L., Imitola J., Deleidi M., Alfaro-Cervello C., Salani G., Porcheri C., Brambilla E., Cavasinni F., Bergamaschi A. (2008). Persistent inflammation alters the function of the endogenous brain stem cell compartment. Brain.

[54] Rasmussen S., Imitola J., Ayuso-Sacido A., Wang Y., Starossom S.C., Kivisakk P., Zhu B., Meyer M., Bronson R.T., Garcia-Verdugo J.M. (2011). Reversible neural stem cell niche dysfunction in a model of multiple sclerosis. Ann. Neurol..

[55] Liu Q., Sanai N., Jin W.N., La Cava A., Van Kaer L., Shi F.D. (2016). Neural stem cells sustain natural killer cells that dictate recovery from brain inflammation. Nat. Neurosci..

[56] Rivera A.D., Butt A.M., Azim K. (2022). Resolving the age-related decline in central nervous system myelin turnover and drug discovery for oligodendroglial rejuvenation. Neural Regen. Res..

[57] Zhang S.C., Ge B., Duncan I.D. (1999). Adult brain retains the potential to generate oligodendroglial progenitors with extensive myelination capacity. Proc. Natl. Acad. Sci. USA.

[58] Rivera A.D., Pieropan F., Williams G., Calzolari F., Butt A.M., Azim K. (2022). Drug connectivity mapping and functional analysis reveal therapeutic small molecules that differentially modulate myelination. Biomed. Pharmacother. = Biomed. Pharmacother..

[59] Nait-Oumesmar B., Picard-Riera N., Kerninon C., Decker L., Seilhean D., Hoglinger G.U., Hirsch E.C., Reynolds R., Baron-Van Evercooren A. (2007). Activation of the subventricular zone in multiple sclerosis: Evidence for early glial progenitors. Proc. Natl. Acad. Sci. USA.

[60] Ekonomou A., Savva G.M., Brayne C., Forster G., Francis P.T., Johnson M., Perry E.K., Attems J., Somani A., Minger S.L. (2015). Stage-specific changes in neurogenic and glial markers in Alzheimer’s disease. Biol. Psychiatry.

[61] James O.G., Selvaraj B.T., Magnani D., Burr K., Connick P., Barton S.K., Vasistha N.A., Hampton D.W., Story D., Smigiel R. (2022). iPSC-derived myelinoids to study myelin biology of humans. Dev. Cell.

